# Eye position affects flight altitude in visual approach to landing independent of level of expertise of pilot

**DOI:** 10.1371/journal.pone.0197585

**Published:** 2018-05-24

**Authors:** David M. Jacobs, Antoine H. P. Morice, Cyril Camachon, Gilles Montagne

**Affiliations:** 1 Facultad de Psicología, Universidad Autónoma de Madrid, Madrid, Spain; 2 Aix-Marseille Université, CNRS, ISM UMR 7287, France; 3 Centre de Recherche de l’Armée de l’Air, Salon de Provence, France; RMIT University, AUSTRALIA

## Abstract

The present study addresses the effect of the eye position in the cockpit on the flight altitude during the final approach to landing. Three groups of participants with different levels of expertise (novices, trainees, and certified pilots) were given a laptop with a flight simulator and they were asked to maintain a 3.71° glide slope while landing. Each participant performed 40 approaches to the runway. During 8 of the approaches, the point of view that the flight simulator used to compute the visual scene was slowly raised or lowered with 4 cm with respect to the cockpit, hence moving the projection of the visible part of the cockpit down or up in the visible scene in a hardly noticeable manner. The increases and decreases in the simulated eye height led to increases and decreases in the altitude of the approach trajectories, for all three groups of participants. On the basis of these results, it is argued that the eye position of pilots during visual approaches is a factor that contributes to the risk of black hole accidents.

## Introduction

Landing is a relatively short flight maneuver during which a comparatively high percentage of fatal and non-fatal aviation accidents occur [1,2]. Perceptual problems contribute importantly to landing phase accidents [3,4]. We focus on a typically ignored aspect of such perceptual problems: altering the visual reference of the cockpit by changing the eye position of the pilot in the cockpit.

Landing can be divided into three phases. In the first phase, the base-to-final turn, the airplane is aligned with the runway. Second, in the approach phase, the pilot gradually decreases speed and altitude in order to maintain a constant glide slope (typically around 3° with respect to the ground) until the airplane reaches the runway. Finally, just before ground contact, the airplane’s nose is tilted slightly upward in a maneuver that is called the landing flare. Our research concerns the approach phase.

### Perceiving the approach angle

The most crucial aspect of the visual control of the approach phase is the perception of the approach angle. The approach angle is the angle between the horizontal ground surface and the line running from the airplane to the aiming point (defined below). Multiple sources of information have been investigated as potential contributors to the perception of this angle [5,6]. A first variable that is typically considered is the length-to-width ratio of the optical projection of the runway, referred to as the *aspect ratio* [6]. If the airplane descends toward the landing location along a constant glide slope (in aviation terminology, along a stabilized glide slope), this ratio has a particular value that depends on the physical length and width of the runway. A higher ratio indicates a higher approach angle and a lower ratio indicates a lower approach angle. This variable therefore allows the pilot to regulate the approach: the correct flight path is followed when the pilot maintains a constant, appropriate aspect ratio.

Consistent with a strategy based on the aspect ratio, it has been shown that larger physical length-to-width ratios of the runway lead to lower approaches, and vice versa [6–8]. The aspect-ratio strategy may be dangerous. This is particularly so for long and narrow runways, because for such runways the predicted approach path is lower than the correct glide path. A study by Gibb and colleagues [9] was based on the observation that the standard Approach Lighting System (ALS), which includes a line of lights before the beginning of the runway, makes the runway look longer and hence affects the perceived aspect ratio. Gibb and colleagues showed that the standard ALS leads to lower flight trajectories. These authors tested a reconfigured ALS, which made the runway appear wider and was hence predicted to lead to higher approach trajectories. Although the reconfigured ALS was only partly successful, the study illustrates that the aspect ratio does play a role in the control process.

A second variable that is commonly associated to the perception of the approach angle is the *absolute H angle* [6,10]. This variable is defined as the angle between the horizon and the aiming point (seen from the pilot’s position). The aiming point is the point on the runway toward which the airplane is progressing during the approach phase. In most cases the airplane lands beyond the aiming point as it covers additional ground during the landing flare. The aiming point coincides with the focus of expansion [11,12]. That is, whereas the optical projection of the aiming point itself does not move, the projections of ground elements in front of, beyond, and to the sides of the aiming point move away from it. The correct flight path is followed when the pilot maintains the absolute *H* angle constant at the appropriate value, while keeping the focus of expansion at the appropriate aiming point.

The absolute *H* angle is based on the horizon as a reference for the direction of the aiming point. The horizon may be difficult to detect, for example during approaches at night, or due to foggy weather. Lintern and Liu [10] provided evidence for the use of the absolute *H* angle by showing that more accurate approaches take place in environments where the horizon could be identified more clearly (either directly or indirectly through perspective lines). Such evidence has led to the suggestion that the absolute *H* angle is “probably the main cue for keeping the glide slope” ([13], p. 13).

The aspect ratio and the absolute *H* angle are often mentioned in flight training. These variables are also described in detail in the Airplane Flying Handbook of the Federal Aviation Administration [14]. With respect to the absolute *H* angle, the handbook states that “for a constant angle glide path, the distance between the horizon and the aiming point remains constant (p. 8–9)”. With respect to the aspect ratio, the handbook states that “during a stabilized approach, the runway shape does not change … if the approach becomes shallow, the runway appears to shorten and become wider. Conversely, if the approach is steepened, the runway appears to become longer and narrower (p. 8–10; cf. Figures 8–10 and 8–11)”.

### The cockpit as visual reference

There are experimental results, however, that cannot be explained by the use of the aspect ratio and the absolute *H* angle. First, in [15], we asked participants to land a virtual airplane using a fixed-base flight simulator, on 245 trials. After a practice phase, participants performed transfer trials in which the cockpit was removed from the visual scene. Without the cockpit, the level of performance fell back to approximately the same level as before the practice phase. Second, in an experiment on variability of practice that used the same flight simulator [16], we applied variability to the simulated height of the participant’s eye with respect to the visible edge of the cockpit (i.e., in the point of view used to compute the visual scene). This variability had a remarkable effect on the flight altitude.

The aspect ratio and the absolute *H* angle can be detected and used without the visual reference of the cockpit. Furthermore, these variables are affected in a negligible way by changes in the eye position in the cockpit. The results reported in [15] and [16] can therefore not be explained by landing strategies based on these traditionally considered variables. Those results indicate that, in addition to the traditionally considered variables, the eye position in the cockpit is related to flight altitude. Because flight altitude can be crucial, for example in black hole accidents [3], the relation between eye position and flight altitude needs to be further investigated.

### Between-trial versus within-trial manipulations

The empirical studies reviewed in the previous sections analyzed modifications in the behavior of participants (e.g., changes in flight trajectories) that followed manipulations of the experimental environment (e.g., changes in the ALS or removing or adding a visible cockpit). The manipulations were applied between trials. An alternative methodology is to examine modifications in behavior when a specific property is gradually modified during the trial. Within-trial manipulations are commonly applied in studies that aim to identify perceptual-motor mechanisms in interceptive tasks [17], heading tasks [18], and locomotor pointing tasks [19]. We believe that within-trial manipulations may also help to identify the perceptual-motor strategies used by pilots. Within-trial manipulations may be more useful than between-trial manipulations if participants would somehow be able to rapidly adapt to changes in the experimental environment at the beginning of a trial.

In the present experiment, then, we modified the simulated eye position of the pilot with respect to the cockpit. As mentioned above, in [16] we also manipulated the eye position of participants who performed the approach phase. Differences between that study and the present one include the facts that the present study (a) used two purposefully chosen levels of the factor eye height rather than this factor being varied randomly as a mere aspect included in a broader study on variability of practice, (b) tested pilots with different levels of expertise in addition to participants without flight experience, and (c) applied within-trial manipulations rather than between-trial manipulations. Our hypothesis is that small variations in the eye height lead to large differences in flight altitude, for all groups of participants.

## Materials and methods

### Ethics statement

This research project was approved by the local institutional review board of the Institute of Movement Sciences (*Comité Ethique de l’Institut des Sciences du Mouvement d’Aix-Marseille Université*). Participants signed an informed consent following the requirements of the Declaration of Helsinki. They were informed of their right to stop their participation at any time.

### Participants

Thirty-two male volunteers participated in the experiment. This population consisted of three subgroups: 10 certified pilots, 10 trainee pilots, and 12 novices. Certified and trainee pilots were recruited from a military airbase at Salon-de-Provence, France. They usually piloted a TB-10 Tobago airplane, similar to the Cessna 172P simulated in the experiment. On average, the certified and trainee pilots had 1990 and 74.1 certified flight hours, respectively. During their training, they had been instructed to use the shape of the runway and the absolute *H* angle in the visual control of the landing phase. Novice participants were recruited from the Faculty of Sport Science in Marseille, France, and had no training or education in aeronautics. All participants had normal or corrected-to-normal vision. They were not informed about the purpose of the study. Further participant information is given in Table 1.

**Table 1:** Age (Years) and flight experience (certified flight hours) of participants.

Novices ^a^	Trainees		Certified Pilots	
Age	Age	Experience	Age	Experience
27	22	25	—^b^	2700
20	22	65	31	1800
21	23	200	30	1300
29	23	150	25	700
22	22	45	30	400
20	22	28	29	1700
19	21	63	35	2600
21	23	45	52	7200
20	22	50	30	1000
—^b^	21	70	25	500
—^b^				
—^b^				

^a^ Novice participants did not have certified flight hours.

^b^ The ages of these participants were not properly registered, but they were similar to the ages of the other participants in their group.

### Apparatus

Participants sat at a distance of 0.6 m from the screen of a laptop (Dell XPS M1730). The screen was 0.36 m wide and 0.24 m high, offering a 36.3 by 26.2° field of view. Participants held an aviation-game joystick (Saitek AV8R) in their preferred hand. The data from the joystick were sent to the laptop via a USB port. These data controlled the elevators of the simulated Cessna 172P. The flight simulator JSBSim [20] was used to compute the flight parameters (pitch, angle of attack, horizontal and vertical position, etc.). The force exerted by the propeller, also controlled by the simulator, was computed so as to propel the airplane at 43.7 m/s when flying parallel to the ground, taking into account the airplane model (e.g., engine number and position, wingspan) and flight conditions (e.g., remaining fuel). The used engine power and the flap settings (no flap extension) led to relatively fast descents. The minimum and maximum speeds registered in the experimental trials were 43.5 and 58.0 m/s, respectively. This means that the airspeed was always above the stalling speed (which, depending on circumstances, is around 25 m/s) and below the maximal structural cruising speed (around 64 m/s). The visual scene was rendered on the laptop screen with ICE (Imagine, Create, & Experiment). ICE is a software package developed at the Institute of Movement Sciences, Marseille. The visual scene was updated at a rate of 60 frames/s.

### Experimental design and procedure

#### Introduction phase

**Fig 1:**
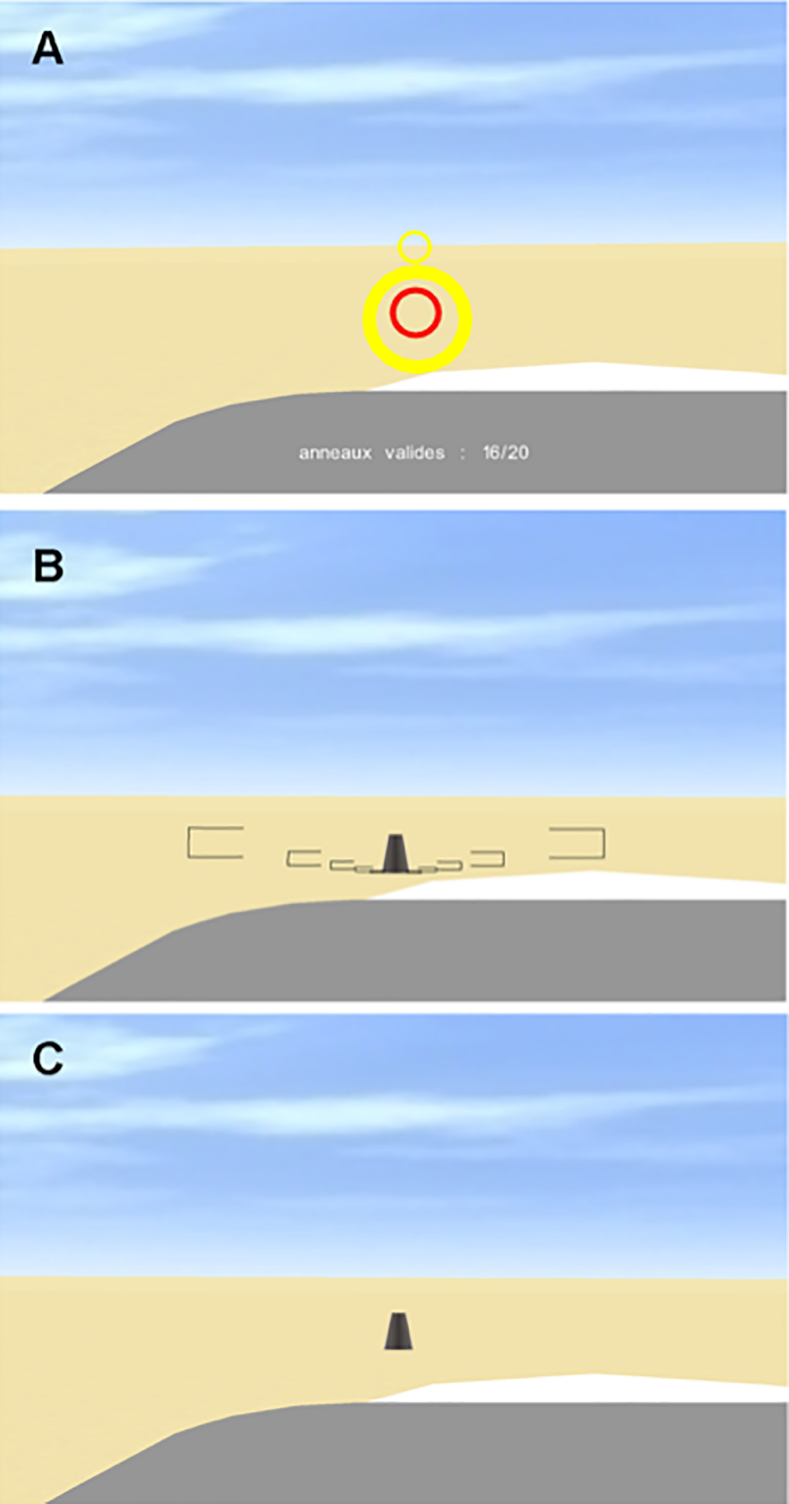
Screenshots of the visual scene. A: introduction phase, B: familiarization phase, and C: experimental phase. The text (in French) that is visible in A indicates the number of rings that have been successfully passed through in the introduction trial.

This phase allowed participants to learn how to use the interface. The visual scene consisted of a Cessna 172P cockpit with no on-board instruments, a yellow sand-like ground surface, light clouds, and 20 rings (Fig 1A; S1 Video). The rings had an inner radius ranging from 4 to 13 m (*M* = 8.4 m; *SD* = 3.4 m) and they were placed at a randomly selected altitude ranging from 190 to 280 m (*M* = 227.8; *SD* = 27.7 m). The horizontal distance between the rings was 100 m. At the beginning of each trial the airplane flew at a speed of 38.6 m/s at an altitude of 189.0 m. Participants were asked to fly through the rings by pushing or pulling the joystick forward or backward from its initial position, hence moving the elevators downward or upward. The introduction phase ended when participants passed through 18 of the 20 rings. All participants achieved this criterion within 5 trials. The introduction phase lasted approximately 15 min.

#### Familiarization phase

This phase was designed to familiarize participants with the experimental task. Participants were asked to visually control the airplane’s altitude in order to maintain the glide slope at 3.71° (for convenience, we use positive values to indicate downward angles, in contrast to the often-used convention to indicate downward angles with negative values). In addition to the ground surface, clouds, and cockpit, the visual scene now also included a virtual runway (2000 m long × 50 m wide). At the start of each trial, the airplane flew at a horizontal distance of 2000 m from the beginning of the runway, at an altitude that was randomly chosen from 34 equidistant values between 121.6 and 131.7 m. This means that the airplane started either below or above the ideal glide slope. During the first second of a trial the autopilot was enabled and the airplane flew parallel to the ground at a speed of 43.7 m/s.

For the rest of the trial, participants controlled the elevators and hence (indirectly) the altitude of the airplane. The lateral position of the airplane remained aligned with the center of the runway. There were 8 familiarization trials. During these trials, as soon as the airplane flew 0.50° above or below the glide slope of 3.71° for more than 2 s, visual brackets indicating the correct glide slope were shown (Fig 1B; S2 Video). In order to compute the glide slope, and for all further analysis, we took the beginning of the runway as the aiming point. The visual brackets disappeared when the airplane was brought back within the reference area (cf. [10]). Trials ended when the airplane was 250 m away from the aiming point. The average trial duration was about 35 s. The familiarization phase lasted approximately 5 min.

#### Experimental phase

The experimental phase consisted of 32 trials: 8 experimental trials with the eye-height manipulation, 20 control trials without manipulation, and 4 familiarization trials with the concurrent feedback in the form of the brackets. The trials in this phase were identical to the ones in the familiarization phase, with the exceptions of the eye-height manipulation in the experimental trials and the absence of the concurrent feedback in the experimental trials and in the control trials (Fig 1C). For completeness, let us mention that the actual experiment also included 8 experimental trials in which the length of the runway was manipulated in a for participants hardly noticeable way. The results for these trials are not reported because (a) the manipulation did not lead to noteworthy results and (b) due to programming error the width of the runway at the further end was modified as well as the length, making the manipulation less interesting.

In the experimental trials with the eye-height manipulation, the vertical position of the point in the cockpit from which the virtual scene was observed was gradually modified. The manipulation started when the airplane was at a horizontal distance of 1000 m from the aiming point and lasted 7 s. The vertical position of the simulated eye varied from an initial position of 0.30 m above the lower edge of the cockpit window to a final position of 0.34 or 0.26 m above that edge. The trials with the increase (S3 Video) and decrease (S4 Video) in eye height were repeated 4 times each. The manipulated eye position is an important parameter in the flight simulator because the visual scene as projected on the screen is computed with regard to this point of observation. Given that the cockpit is much closer to the pilot’s eye position than the outside environment, changing the simulated eye position causes a slow movement of the cockpit along the screen, as well as a more subtle modification of the visible part of the nose of the airplane. Participants were not informed about the experimental manipulation.

The 8 experimental trials with the eye-height manipulation and the 20 control trials were performed in a random order. The 4 familiarization trials were included with the aim of helping participants to maintain an appropriate calibration. Two of these trials were performed after approximately one third of the experimental phase and the remaining two after approximately two thirds of the experimental phase. The experimental phase lasted approximately 25 min. Participants were not asked to indicate how they maintained the appropriate glide slope.

### Analysis

The analyses addressed the flight altitudes and the approach angles that were computed from the flight altitudes. All analyses were run on the individual averages per experimental condition. In other words, for each participant we averaged the flight altitudes and approach angles over the 20 control trials and over the 4 experimental trials with the increase and decrease in eye height, respectively. The flight altitudes in the individual trials can be found in the supporting information (S1 Dataset). Huynh-Feldt adjustments of *p* values and of degrees of freedom were reported whenever the sphericity assumption was violated in analyses of variance (ANOVAs).

## Results

This Results section consists of two subsections. The first one inspects performance in the control trials. Although the analyses in this first subsection did not reveal significant differences among the groups with different levels of expertise, they allow the reader to obtain an overall impression of performance. The second subsection, on performance in the experimental trials, addresses the effect of simulated eye position on flight altitude.

### Performance in control trials

**Fig 2:**
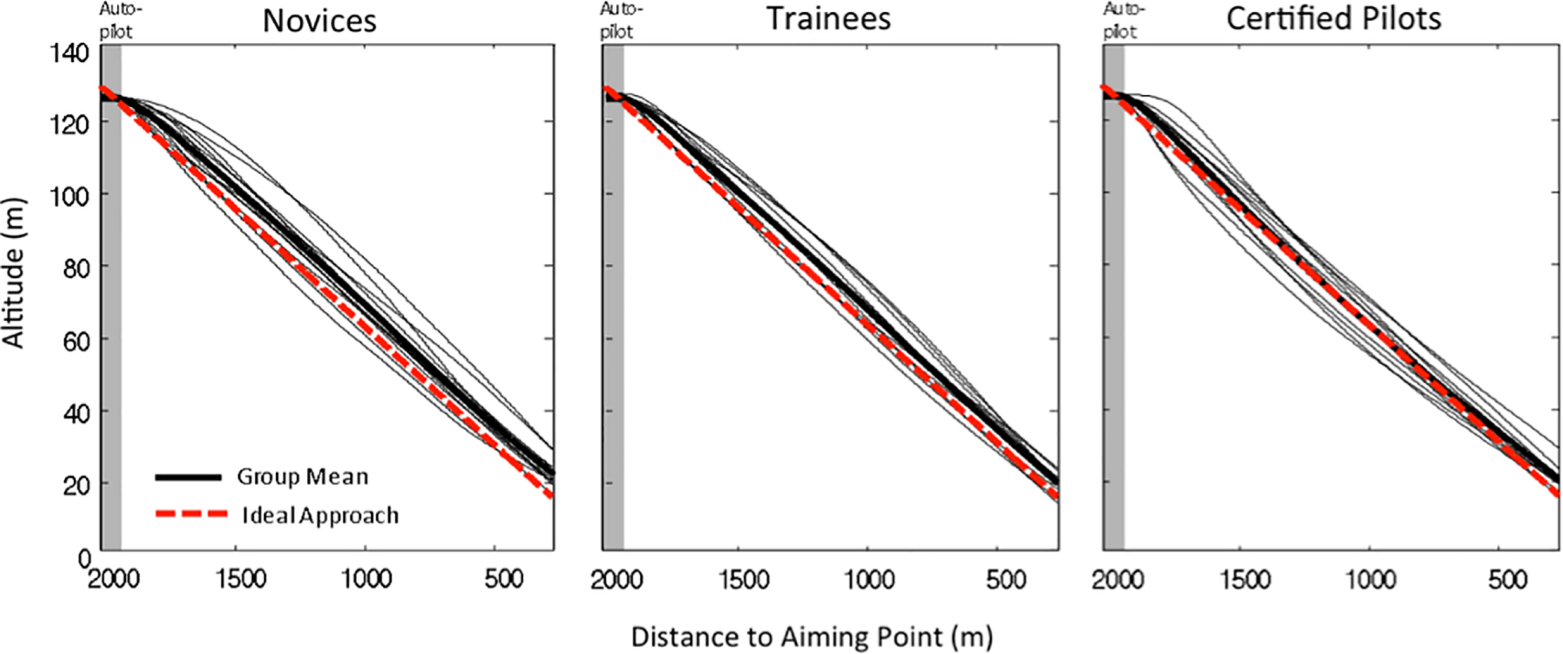
Flight altitudes in control trials. Group averages (thick curves) and individual averages (thin curves) of the flight altitudes, as a function of the distance to the aiming point, for novices, trainees, and certified pilots. The dashed red line indicates the altitude corresponding to the ideal 3.71° approach angle. The gray area indicates the autopilot period.

Fig 2 shows the flight altitudes as a function of the distance to the aiming point, for the novices, trainees, and certified pilots. The thick black line, indicating the overall group average, seems to lie slightly higher above the dashed red line for the novices (left panel) and the trainees (middle panel) than for the certified pilots (right panel). For the certified pilots, the thick black line and the dashed red line largely overlap, indicating that the average flight trajectory of the certified pilots coincided with the ideal glide slope for substantial parts of the approach. Averaged over the full approach, the distance between the flight trajectories and the ideal trajectory was 5.3 m for the novices, 3.8 m for the trainees, and 1.6 m for the certified pilots. At 1000 m before the aiming point, this distance was 6.14 m for the novices, 4.32 m for the trainees, and -0.03 m for the certified pilots.

To test the differences in flight altitude, we performed an ANOVA with a split-plot design (see p. 593 of [21]). Expertise (novices, trainees, certified pilots) was used as a between-subjects factor and distance to the aiming point (from 2000 to 250 m in intervals of 250 m) as a within-subjects factor. The effect of distance was significant, *F*(2.5, 71.2) = 5011.1, *p* < .001, η^2^_*p*_ = 0.99, indicating the obvious fact that participants descended when they approached the runway. The effect of expertise, *F*(2, 29) = 2.1, *p* = .14, and the interaction, *F*(4.9, 71.2) = 1.8, *p* = .12, were not significant. Hence, the ANOVA did not confirm that the certified pilots flew closer to the ideal trajectory. As a further test of the group differences in flight altitude, we performed a one-way ANOVA with expertise (novices, trainees, certified pilots) as factor on the distance between the individual-average flight trajectories and the ideal flight trajectory at 1000 m before the aiming point. This one-way ANOVA revealed only a non-significant tendency, *F*(2, 29) = 2.8, *p* = .078.

**Fig 3:**
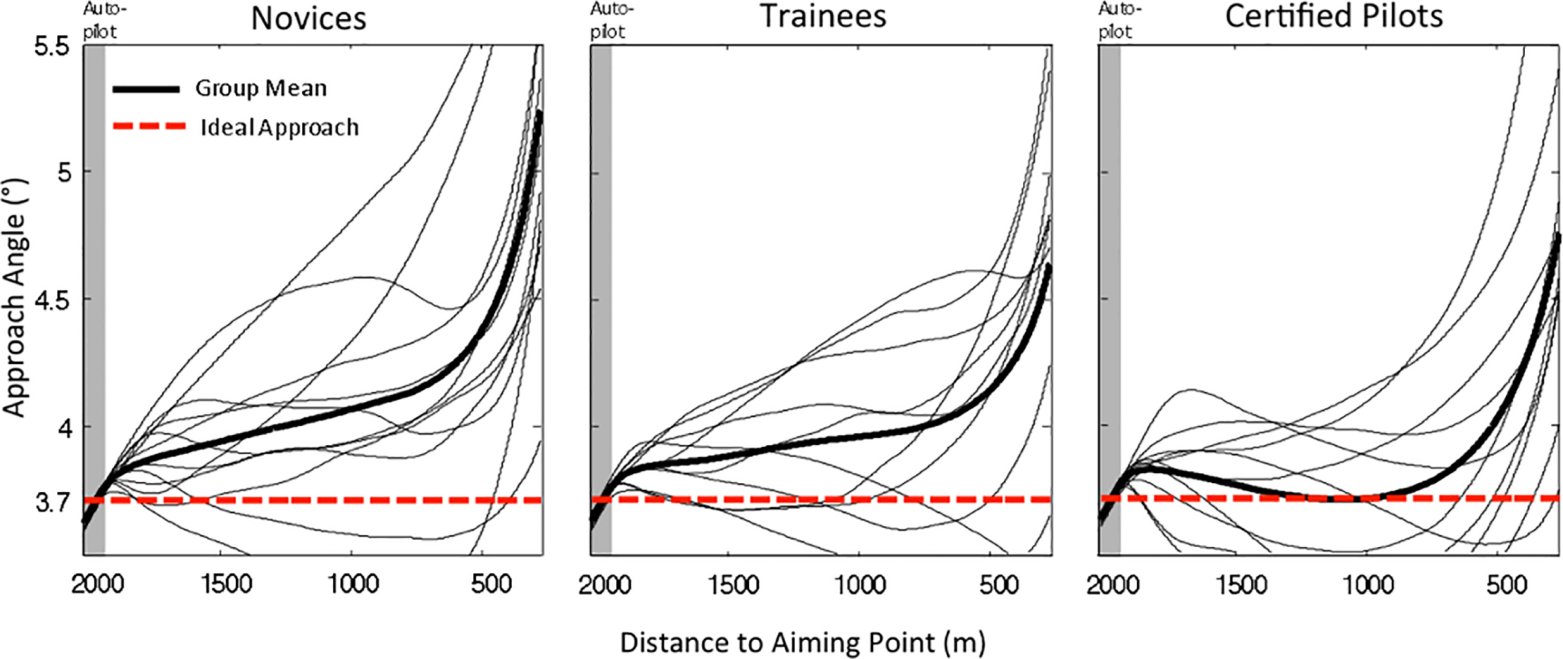
Approach angles in control trials. Group averages (thick curves) and individual averages (thin curves) of the observed approach angles as a function of the distance to the aiming point, for novices, trainees, and certified pilots. The dashed red line indicates the ideal 3.71° approach angle.

Fig 3 shows the approach angles for the three groups. As for the flight altitudes, we performed a split-plot ANOVA on the approach angles with expertise (novices, trainees, certified pilots) as a between-subjects factor and distance to the aiming point (from 2000 to 250 m in intervals of 250 m) as a within-subjects factor. The effect of distance was significant, *F*(1.5, 44.8) = 51.1, *p* < .001, η^2^_*p*_ = 0.64, reflecting an increase in approach angle when the participants got closer to the aiming point. In part this is so because flying a few meters above the ideal glide slope has a larger effect on the approach angle when one is closer to the aiming point. The effect of expertise, *F*(2, 29) = 2.0, *p* = .15, and the interaction, *F*(3.1, 44.8) = 1.7, *p* = .19, were not significant. To summarize, although certified pilots seemed to fly slightly lower and with approach angles nearer to the stipulated value of 3.71°, our analyses on the control trials did not reveal significant differences among the groups with different levels of expertise.

### Performance in experimental trials

This subsection addresses performance in the trials in which the height of the simulated eye position increased or decreased by 4 cm during the 7-s interval that started at 1000 m. With respect to these trials, we first report a small but significant effect at the onset of the manipulation.

At 1000 m before the aiming point, the averaged approach angles in the trials with increases and decreases in eye height were 3.94 and 3.85°, respectively. To test this difference, we performed an ANOVA with a split-plot design on the individual mean approach angles at 1000 m. Expertise (novices, trainees, certified pilots) was used as a between-subjects factor and eye height (increase, decrease) as a within-subjects factor. The effect of eye height was significant, *F*(1, 29) = 5.7, *p* = .024, η^2^_*p*_ = 0.16. The effect of expertise, *F*(2, 29) = 1.5, *p* = .25, and the interaction, *F*(2, 29) = .2, *p* = .82, were not significant. Given that an effect of eye height was hypothesized to occur after the onset of the manipulation, observing the effect before the manipulation is for methodological reasons undesirable. We therefore compensated the effect by subtracting 0.05° from all approach angles in the increase condition and adding 0.05° to all approach angles in the decrease condition, from the beginning to the end of each trial. This made the overall averages of the approach angles at the onset of the manipulation identical in the different conditions.

**Fig 4:**
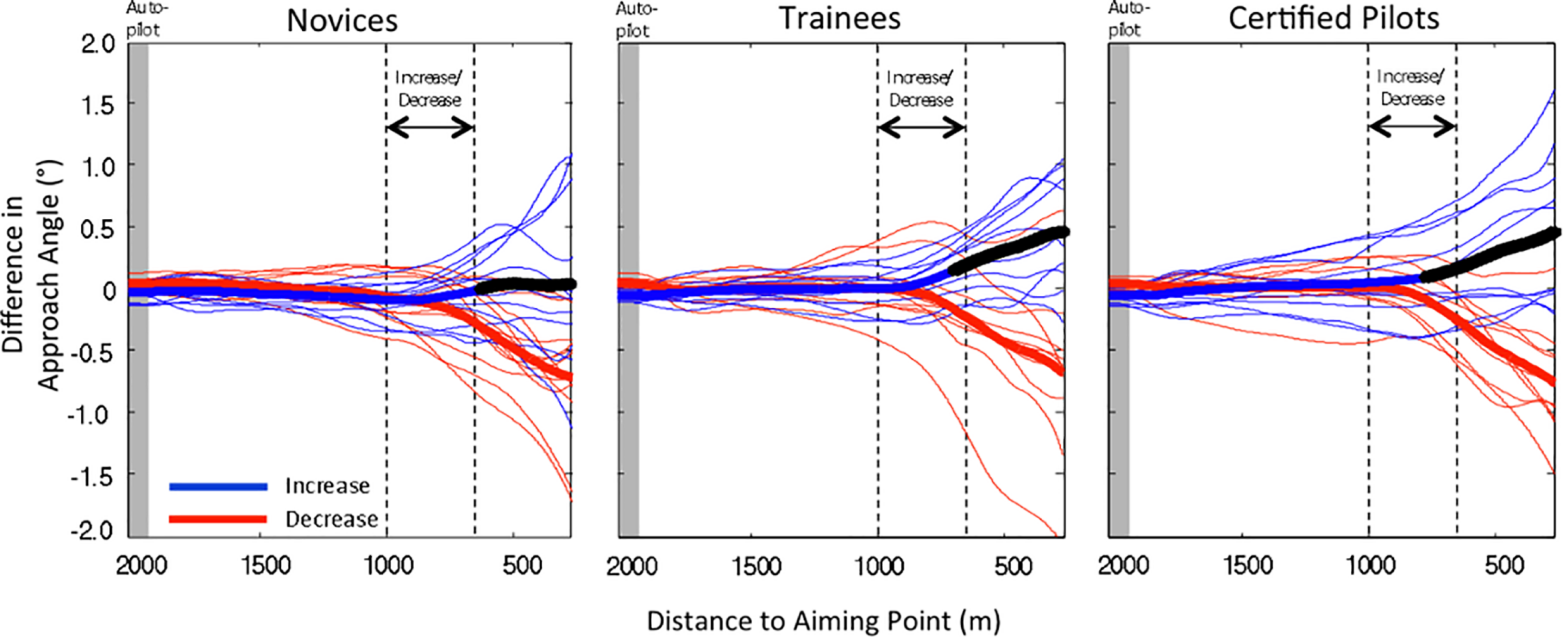
Approach angles in the experimental trials. Group averages (thick curves) and individual averages (thin curves) in the increase condition (blue curves) and in the decrease condition (red curves), for novices, trainees, and certified pilots. The curves show the approach angles as compared to those in the control trials. The vertical dashed lines indicate the beginning and the end the manipulation of the simulated eye height. Asterisks on the thick blue curves indicate that, at those points, single-tailed *t* tests comparing the corrected individual-average approach angles in the increase and decrease conditions were significant (*p* < .05).

Fig 4 presents the approach angles in the increase (blue curves) and decrease (red curves) conditions. To better appreciate the effect of the manipulation, the curves show the difference in the approach angles with respect to those observed in the control condition. Due to the correction referred to in the previous paragraph, the overall average heights of the blue and red curves at 1000 m are identical. The divergence of the thick blue and red curves after the onset of the manipulation indicates that participants showed larger approach angles in the increase condition than in the decrease condition. A split-plot ANOVA on the (corrected) approach angles at 250 m before the aiming point, with expertise (novices, trainees, certified pilots) as a between-subjects factor and eye height (increase, decrease) as a within-subjects factor, revealed a significant effect of eye height, *F*(1, 29) = 53.5, *p* < .001, η^2^_*p*_ = 0.65. The effect of expertise, *F*(2, 29) = 0.7, *p* = .51, and the interaction, *F*(2, 29) = 1.1, *p* = .36, were not significant.

As an alternative to the compensation used in the previous paragraph, pre-manipulation effects can be compensated with an analysis on gain scores computed from the onset of the manipulation to the end of the trial (see p. 444 of [21]). In addition to the analyses reported in the previous paragraph, we therefore performed a split-plot ANOVA on the (uncorrected) change in approach angle from 1000 to 250 m. The results of this analysis replicated the results reported in the previous paragraph. The effect of the within-subjects factor eye height was significant, *F*(1, 29) = 75.2, *p* < .001, η^2^_*p*_ = 0.72, showing that the gain in approach angle was larger in the condition with an increase in eye height. The effect of expertise, *F*(2, 29) = 0.98, *p* = .39, and the interaction, *F*(2, 29) = 1.2, *p* = .32, were not significant.

The difference in altitude in the increase and decrease conditions at 250 m from the aiming point was 4.92 m. At the onset of the manipulation, this difference was 1.68 m. If one corrects the altitude at the end of the trial for the difference at the onset of the manipulation, a difference of 3.25 m is obtained that is attributable to the manipulation. The average altitude at 250 m was 20.5 m. The corrected difference between the eye height conditions at the end of the trial therefore represents 15.8% of the average flight altitude.

## Discussion

In the presented experiment, three groups of participants with different levels of expertise were asked to maintain a constant glide slope during the approach to landing. The simulated eye position with respect to the cockpit was manipulated during the approach (i.e., within the trials). Independent of the level of expertise, raising the eye led to higher approaches and lowering the eye led to lower approaches. The demonstrated importance of the eye position relative to the cockpit is consistent with previous results obtained with novice participants and with between-trial manipulations [15,16].

### Informational variables implied in the control

The effect of eye height cannot be explained by the exclusive use of the aspect ratio and the absolute *H* angle. This is so because these commonly considered variables are not affected by minor changes in the position of the eye in the cockpit. Other informational variables must therefore be implied in the control of the final approach—most probably in addition to the aspect ratio and the absolute *H* angle.

One candidate variable is the relative *H* angle [6,16]. This variable is defined as the angle between the aiming point and an arbitrary point on the airplane, such as a point on the lower edge of the cockpit window. Although it does not explicitly refer to this variable, the Airplane Flying Handbook of the Federal Aviation Administration mentions that “During the approach, round out, and touchdown; vision is of prime importance … Visual focus is not fixed on any one side or any one spot ahead of the airplane. Instead, it is changed slowly from a point just over the airplane’s nose to the desired touchdown zone and back again (p. 8–5 of [14]; see [22–24] for actual eye-registration results).” The eye movements recommended in this quote may be interpreted as facilitating the detection of the relative *H* angle.

It is easy to relate the relative *H* angle to our experimental results. If eye height is increased, the visible parts of the cockpit and the nose of the airplane move down with respect to the rest of the visual scene, including the projection of the aiming point. This implies an increase in the relative *H* angle. The increase in relative *H* angle can be cancelled by a corresponding increase in flight altitude. Likewise, a decrease in eye height leads to a decrease in the relative *H* angle, which can be cancelled by a decrease in altitude. The effect of eye height observed in our experiment is hence consistent with a strategy in which the pilot modifies the flight trajectory so as to cancel changes in the relative *H* angle.

The use of the relative *H* angle in the control of the final approach, however, is not as straightforward as the use of the aspect ratio and the absolute *H* angle. In contrast to the other variables, the relative *H* angle depends on the pitch of the airplane: if the airplane turns nose-down, the relative *H* angle increases, and if it turns nose-up, the relative *H* angle decreases. In other words, the relative *H* angle is not specific to the approach angle. Without further assumptions, it can therefore not be considered as a substitute for the aspect ratio or the absolute *H* angle.

One may also speculate that the manipulation of eye height was effective because it affected variables that are used for the perception of the pitch or the altitude of the airplane. A candidate variable for the perception of pitch is the angle between the horizon and an arbitrary point on the airplane (see Figure 3–8 in [14]). This variable is biased by increases and decreases in eye height in a similar way as the relative *H* angle. If pilots perceive the pitch of the airplane on the basis of this variable, changes in eye height should be expected to affect the pitch control and, as a consequence, the flight altitude. This means that the use of such variables may also be consistent with our results.

### Eye position and posture

Independent of the open questions concerning the use of informational variables, if one assumes that our findings generalize to real environments, they would have important consequences. In real environments, small changes in eye position are common. Changes in eye position may be due, for example, to changes in posture from one day to another, or from one moment to another, or to the use of adjustable seats or cushions. Our study indicates that such small changes in eye position may lead to substantial changes in flight altitude: moving the simulated eye 4 cm up or down led to an average difference of 15.8% in altitude.

The Airplane Flying Handbook of the Federal Aviation Administration [14] warns for postural effects in the section on straight-and-level flight. It states that “straight-and-level flight is a matter of consciously fixing the relationship of a reference point on the airplane in relation to the natural horizon (p. 3–7)” and that “the reference point depends on the pilot’s seating position, height, and manner of sitting (p. 3–7)”. We believe that the visual reference of the cockpit is crucial also in the approach to landing, and, hence, that a similar warning would be appropriate for the approach phase.

### Implications: Black hole accidents

Most critically, changes in the position of the eye—or in the reference point due to, say, flying a different airplane or forgetting the exact reference point that was used—may be related to black hole accidents. Black hole accidents occur under circumstances with a reduced visibility of terrain features other than the runway, for instance at night. Under such circumstances low approaches have been reported and airplanes sometimes hit the ground before reaching the runway [3].

As many other types of accidents, black hole accidents have been argued to require an unfortunate combination of multiple contributing factors (see p. 38 of [4]). Our results indicate that, in this sense, sitting posture has the potential to be the straw that breaks the camels back. A less upright posture of the pilot, perhaps as a result of the fatigue associated to a night flight, may lower the eye position. This may lead to a yet lower approach that, due to the lack of visibility, may go undetected. Being aware of this contributing factor—and sitting more straight up—may reduce the risk of fatal consequences.

## Conclusions

This study tested the hypothesis that minor changes in eye height affect the flight altitude during the final approach to landing. The hypothesis was confirmed. In addition to raising awareness about the importance of eye height, our findings may inspire further research. Research with flight simulators could address, for instance, how the pattern of fixation of pilots may be affected by changes in the location of the point of view relative to the edge of the cockpit window (cf. [22–24]). Above all, however, it would be interesting to monitor if naturally occurring (or experimentally induced) changes in eye position in real-life situations co-vary with modifications of flight trajectories.

## Supporting information

S1 Dataset Flight altitudes in experimental and control trials, sampled every 10 m. (ZIP)

S1 Video Introduction trial. (GIF)

S2 Video Familiarization trial. (GIF)

S3 Video Experimental trial with increase in eye height. (GIF)

S4 Video Experimental trial with decrease in eye height. (GIF)
