# Supplementary figures and images for: Eye position affects flight altitude in visual approach to landing independent of level of expertise of pilot
Source: PLoS One. 2018 May 24;13(5):e0197585. doi: 10.1371/journal.pone.0197585 (PMC5967751; doi:10.1371/journal.pone.0197585)

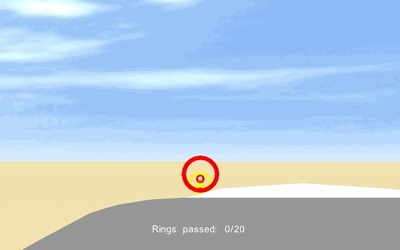

Supplement: S1 Video — (GIF) [file pone.0197585.s002.gif]

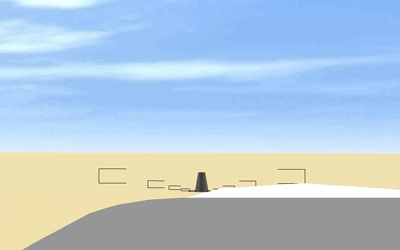

Supplement: S2 Video — (GIF) [file pone.0197585.s003.gif]

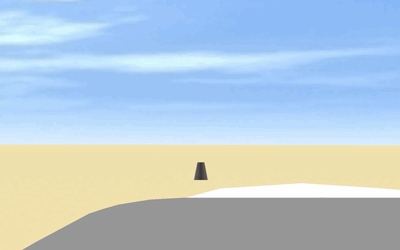

Supplement: S3 Video — (GIF) [file pone.0197585.s004.gif]

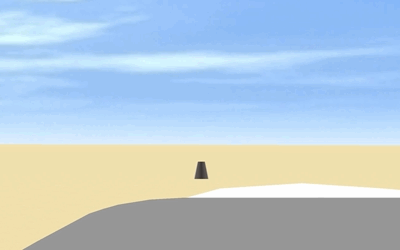

Supplement: S4 Video — (GIF) [file pone.0197585.s005.gif]
